# Neurological Symptoms and Complications of COVID-19 Among Patients in a Tertiary Hospital in Saudi Arabia

**DOI:** 10.7759/cureus.19200

**Published:** 2021-11-02

**Authors:** Abdullah A Tawakul, Ahmad H Alharbi, Ahaad M Basahal, Abdulrahman M Almalki, Bashaer Alharbi, Murouj Almaghrabi, Ahmad Imam

**Affiliations:** 1 Neurology, Faculty of Medicine, Umm Al-Qura University, Al-Abdia Main Campus, Makkah, SAU; 2 Department of Medicine and Surgery, College of Medicine, Umm Al-Qura University, Al-Abdia Main Campus, Makkah, SAU; 3 Internal Medicine-Endocrinology, Diabetes and Metabolism, Faculty of Medicine, Umm Al-Qura University, Makkah, SAU

**Keywords:** saudi arabia, stroke, neurologic manifestations, sars-cov-2, covid-19

## Abstract

Objectives

In this study, we aimed to determine the frequency of neurological signs, symptoms, and complications in coronavirus disease 2019 (COVID-19) patients. We also sought to explore the general characteristics of stroke patients in particular.

Methods

A retrospective cohort study was conducted among COVID-19 patients who were hospitalized between April-September 2020 at the Al-Noor Specialist Hospital in Makkah city, Saudi Arabia. The study included patients who were aged ≥18 years and presented with or were reported to have any neurological manifestations and/or complications secondary to COVID-19 infection.

Results

A total of 79 patients were included. The mean age of the cohort was 63.6 years, with a significant male predominance (67.1%). The most commonly reported neurological signs and symptoms were altered level of consciousness (45.9%), dizziness (11.5%), and focal neurological deficit (10.4%). Acute ischemic stroke was seen in 18 patients. Most of them were males (66.7%). Most strokes were in the right middle cerebral artery territory (MCA) (50.0%). Diabetic patients were four times more at risk to develop stroke [odds ratio (OR)=3.76; 95% confidence interval (CI): 1.1-29.9]. Patients with respiratory failure were 21 times more likely to have a stroke (OR=21.3; 95% CI: 2.2-54.6). Patients with acute respiratory distress syndrome recorded a three-fold increased risk for developing stroke (OR=2.96; 95% CI: 1.25-37.3). Critically ill patients had double the risk of stroke (OR=1.8; 95% CI: 1.1-6.9). Other neurological complications were hemorrhagic stroke (3.3%), subacute/chronic infarction (23.3%), meningitis (10%), and brain mass lesion (3.3%).

Conclusions

Neurological symptoms and complications are not uncommon among COVID-19 patients. Most of these patients had poor outcomes. Acute ischemic stroke was the most common finding on neuroimaging.

## Introduction

Coronavirus disease 2019 (COVID-19) is one of the coronavirus (CoV) outbreaks that is caused by severe acute respiratory syndrome coronavirus 2 (SARS-CoV-2) [1]. According to the literature, the common symptoms at the onset of COVID-19 illness are fever, cough, shortness of breath, sore throat, fatigue [2,3], diarrhea, and nausea/vomiting, while some patients are asymptomatic [4]. In severe cases, patients may develop pneumonia, acute respiratory distress syndrome (ARDS), acute cardiac problems, and multiple organ failure [5], and chest CT scan findings have revealed the clinical features of pneumonia [4].

Recent studies have revealed that SARS-CoV-2 also has neuro-invasive abilities and could transmit from the respiratory system to the central nervous system; the most commonly reported symptoms are headache, hyposmia, dizziness, impaired consciousness, acute cerebrovascular disease, epilepsy, ataxia, acute disseminated encephalomyelitis (ADEM), and viral encephalitis [4]. A study showed that neurological sequelae were seen in 36.4% of the patients, while 5.7% of them had acute cerebrovascular accidents (CVA) [6]. Hence, acute cerebrovascular disease is one of the most common and serious neurologic complications seen in COVID-19 patients [7].

Based on our review of the literature, several studies have reported the development of stroke in COVID-19 patients [5,6,8-10], and the incidence of stroke in COVID-19 patients ranges from 2.5 to 6% [11]. The potential for stroke involvement in COVID-19 is a matter of grave concern. However, local studies on Arab populations, specifically in the Kingdom of Saudi Arabia (KSA), have been scarce. In light of this, the present study aimed primarily to determine the frequency of neurological signs, symptoms, and complications in COVID-19 patients at the Al-Noor Specialist Hospital, Makkah, Saudi Arabia. We also aimed to explore the general characteristics of stroke patients in particular.

## Materials and methods

Study design 

For the current study, we employed a retrospective cohort study design to collect the relevant from the medical records of the Al-Noor Specialist Hospital in Makkah city, Saudi Arabia. 

Sample population

**Figure 1:**
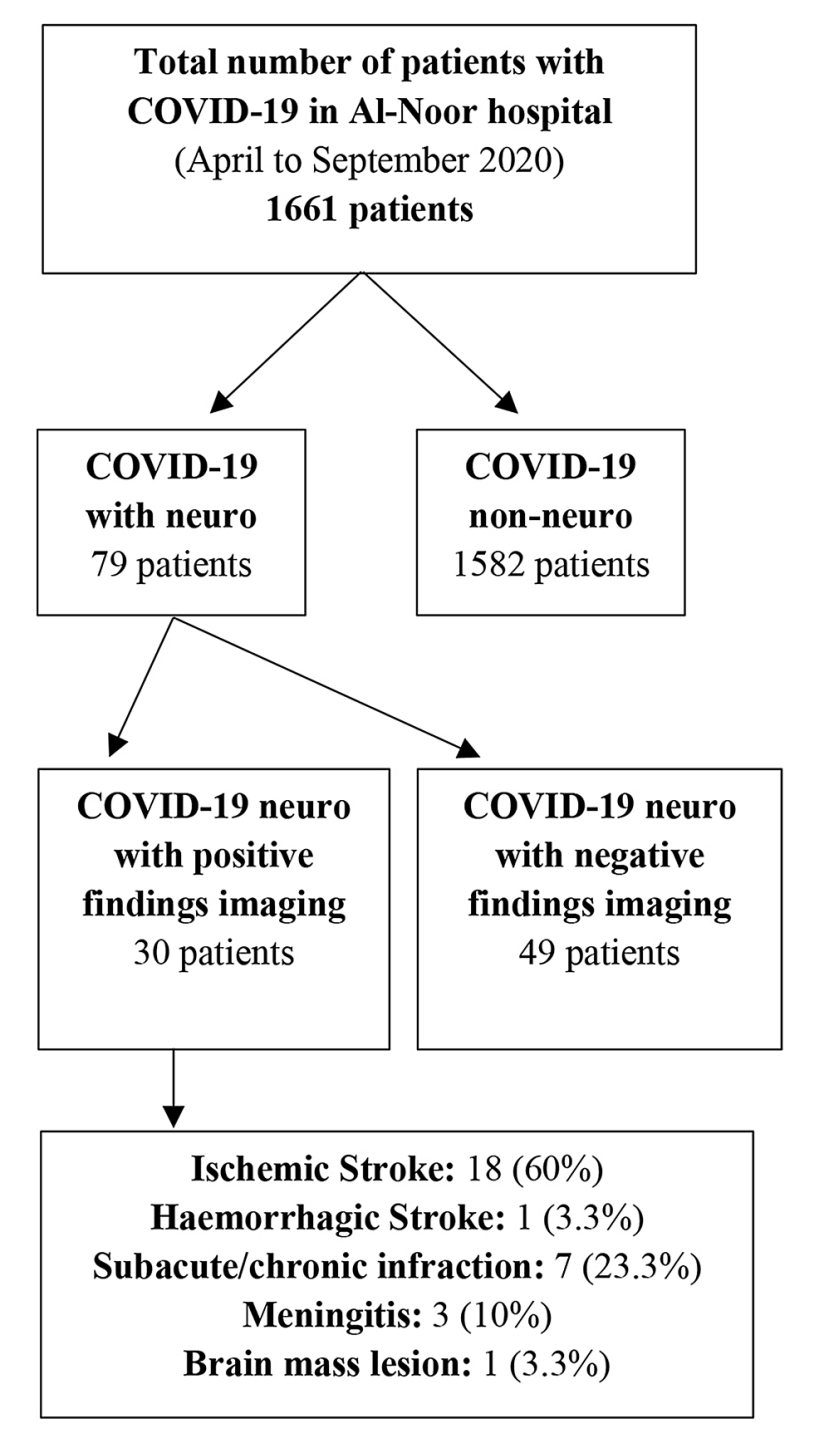
Flow chart summarizing the characteristics of the study sample COVID-19: coronavirus disease 2019

The study’s sample included a final tally of 79 patients (Figure 1). All patients were admitted to the hospital during the period from April to September 2020 during the ongoing COVID-19 pandemic. Our eligibility criteria were as follows: all patients hospitalized at the Al-Noor Specialist Hospital who were laboratory-confirmed to have COVID-19 infection by a nasopharyngeal or oropharyngeal swab specimen with a positive result on real-time reverse transcriptase-polymerase-chain-reaction (RT-PCR) assay [12]. In addition, all included patients presented with neurological signs and symptoms or were reported to have some kind of neurological complication. Patients younger than 18 years in age were excluded from the study.

Recruitment 

A non-probability consecutive sampling technique was used in the current study. Five data collectors collected the required data based on predefined criteria from the patients’ files through an online Google Sheet. Further, the admission registry system of the Al-Noor Specialist Hospital was reviewed to collect the remaining data (e.g., brain CT scans). The Al-Noor Specialist Hospital was chosen as the setting of the study since it serves the citizens, residents, and pilgrims in the Holy Makkah city and its surrounding areas. The hospital is located in the heart of the city near the holy sites (Muzdalifa), at a distance of 3.5 kilometers from the Grand Mosque. It is an approximately 500-bedded facility and provides tertiary care for COVID-19 patients [13]. Additionally, during the first half of the year 1442 Hijri, the hospital served 58,028 emergency cases and performed 4,113 surgeries [14].

Data collection tool

The data collected on the online Google Sheet were divided into three main sections: 1) sociodemographic characteristics and general data of the patients that included age, gender, nationality, admission location (whether in the medical ward or the ICU), hospitalization period, and any known comorbidities. 2) Patients’ current reports including reported neurological signs and symptoms, reported acute cerebrovascular complications, ventilation requirement, laboratory findings, outcomes (mortality, transition to other healthcare settings, improved or stable clinical condition), and the severity level of COVID-19 that was measured based on the National Institutes of Health (NIH) guidelines [15]. As per NIH guidelines, the severity of the illness was categorized into five types according to the clinical presentation of the patients as follows: asymptomatic or pre-symptomatic infection, mild illness, moderate illness, severe illness, and critical illness. 3) Specific data for patients who were diagnosed with stroke including the type of stroke, location of the stroke, lesion side, CT brain findings, and outcomes assessed using the Modified Rankin Score (mRS), which is a six-point scale that is widely used to measure the degree of disability or reliance in daily activities in patients with stroke [16,17].

Statistical analysis

All collected data were transferred from the online Google Sheet onto a Microsoft Excel spreadsheet and then to SPSS Statistics version 23.0 (IBM, Armonk, NY) to perform the statistical analysis. Categorical variables were expressed as frequencies (n) and percentages (%), while numerical data were presented as mean ± standard deviation (SD). Categorical variables were compared using the χ2 test. Two-tailed tests with an alpha error of 0.05 were used. Multivariate analyses were performed using a logistic regression model to adjust for factors associated with stroke among COVID-19 patients. The confidence interval (CI) was set at 95%, and the statistical significance was set at p<0.05.

Ethical considerations

The present study was approved by the Institutional Review Board (IRB) of the Umm Al-Qura University (UQU), Makkah, Saudi Arabia (approval no. 439). Since this was a retrospective study, the requirement for patient consent was waived.

## Results

Sociodemographics and general characteristics

Among a total of 1,661 patients confirmed to have COVID-19 infection at the Al-Noor Specialist Hospital, only 79 patients had neurological manifestations and hence were enrolled in the present study. The mean age of the patients was 63.6 years (SD 13.9; range: 18-95 years). There was a significant male predominance in the cohort (53, 67.1%). The most common nationalities were Saudis (43, 54.4%), followed by patients from Myanmar (nine, 11.4%), and Yemeni (six, 7.6%). Most patients had diabetes mellites (DM) (60, 75.9%) and hypertension (HTN) (58, 73.4%). Self-reported and clinically verified symptoms of COVID-19 were as follows: fever (49, 62%), shortness of breath (47, 59.5%), and cough (40, 50.6%). Additional information on the demographic characteristics of the patients is presented in Table 1.

**Table 1:** Sociodemographics and general characteristics of the study population (n=79) *Include one patient each of the following nationalities: Burkina Faso, Afghani, Egyptian, Indian, Mali, Moroccan, Syrian, and Tunisian; **COPD, SCA, PAD, CAD, leukemia, sepsis, cancer, hypothyroidism, pulmonary edema, and cardiac diseases; ***data related to seven patients missing SD: standard deviation; ICU: intensive care unit; TIA: transient ischemic attack; MI: myocardial infarction; IHD: ischemic heart disease; BMI: body mass index; A-fib: atrial fibrillation; ARDS: acute respiratory distress syndrome; COVID-19: coronavirus disease 2019; COPD: chronic obstructive pulmonary disease; SCA: sudden cardiac arrest; PAD: peripheral arterial disease; CAD: coronary artery disease

Variable	Values
Age (years), mean ± SD	63.6 ± 13.9
Gender, n (%)	
Male	53 (67.1)
Female	26 (32.9)
Nationality, n (%)	
Saudi	43 (54.4)
Myanmar	9 (11.4)
Yemeni	6 (7.6)
Bangladeshi	5 (6.3)
Nigerian	4 (5.1)
Pakistani	2 (2.5)
Chinese	2 (2.5)
Others*	8 (10.1)
Admission location, n (%)	
ICU	52 (65.8)
Medical ward	27 (34.2)
Hospitalization period (days), mean ± SD	12.4 ± 10.8
Known comorbidities, n (%)	
Diabetes mellitus	60 (75.9)
Hypertension	58 (73.4)
Kidney diseases	29 (36.7)
Prior stroke, TIA, or MI	23 (29.1)
IHD	12 (15.2)
Obesity (BMI >30)	9 (11.4)
A-fib	6 (7.6)
Asthma	5 (6.3)
Others**	40 (50.6)
Complications developed during hospital admission, n (%)	
Respiratory failure	17 (21.5)
Septic shock	28 (35.4)
ARDS	12 (15.2)
General symptoms of COVID-19, n (%)	
Fever	49 (62)
Shortness of breath	47 (59.5)
Cough	40 (50.6)
Fatigue	7 (8.9)
Vomiting	6 (7.6)
Diarrhea	4 (5.1)
Nausea	2 (2.5)
Outcome, n (%)***	
Alive	23 (29.1)
Dead	42 (53.2)
Referred to another center	7 (8.9)

**Figure 2:**
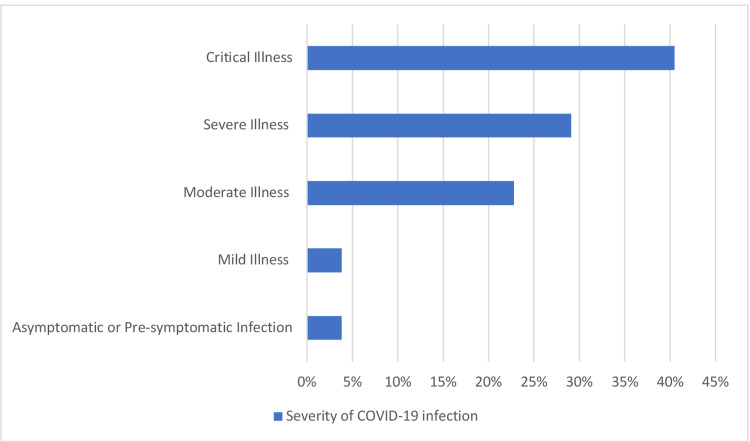
Severity of COVID-19 infection among patients with neurological symptoms or complications (n=79) COVID-19: coronavirus disease 2019

The severity of the COVID-19 infection among the patients is illustrated in Figure 2. Most patients had critical illness (32, 40.5%), followed by patients with severe illness (23, 29.1%), and moderate illness (18, 22.8%). Patients with mild illness and asymptomatic or pre-symptomatic illness were equally represented (three, 3.8% for each group).

Frequent neurological symptoms among COVID-19 patients 

The most frequently reported symptoms among the included patients were altered level of consciousness (44, 45.9%), dizziness (11, 11.5%), focal neurological deficit (10, 10.4%), headache (four, 4.2%), and lastly, syncope/fall (one patient, 1.0%) (Table 2).

**Table 2:** Frequent neurological symptoms among COVID-19 patients (n=79) COVID-19: coronavirus disease 2019

Symptom	Total			Asymptomatic, mild, or moderate illness			Severe illness			Critical illness	
	N	%		N	%		N	%		N	%
Headache	4	4.2		3	75.0		0	0.0		1	25.0
Dizziness	11	11.5		5	45.5		4	36.4		2	18.2
Altered level of consciousness	44	45.9		10	22.7		16	36.4		18	40.9
Syncope/fall	1	1.0		0	0.0		0	0.0		1	100.0
Focal neurological deficit	10	10.4		2	20.0		2	20.0		6	60.0

Neuroimaging findings

Among the 79 patients included in the study, 30 had acute cerebrovascular complications or positive imaging findings; 18 patients had ischemic stroke, one patient had hemorrhagic stroke, seven patients had subacute/chronic infraction, three patients had meningitis, and one patient had a brain mass lesion.

Table 3 presents a comprehensive summary of the clinical characteristics of COVID-19 patients with ischemic and hemorrhagic stroke (n=19). More than half of the stroke patients were male (12, 66.7%). Saudis (nine, 50%) and non-Saudis (nine, 50%) were equally represented. The most commonly reported comorbidities were DM (16, 88.9%) and HTN (13, 72.2%), while a few patients had a prior history of stroke or transient ischemic attack (TIA) (five, 27.8%). Most patients had a severe and critical illness of COVID-19 (38.9% and 33.3%, respectively) and consequently, patients who required mechanical ventilation due to their condition were in the majority (72.2%). Regarding the location of the stroke, the majority were in the middle cerebral artery (MCA) (nine, 50.0%), followed by posterior cerebral artery (PCA), and basilar artery (three, 16.7% each for the two groups). The mRS score was 6 in half of the patients (nine, 50.0%), while three patients (16.7%) had a score of 0-2. More than half of the patients died during hospitalization (11, 61.1%), while three patients had better outcomes and were discharged home (three, 16.7%), and four patients were referred to another center (four, 22.2%).

**Table 3:** Clinical characteristics of COVID-19 patients with ischemic and hemorrhagic stroke (n=18) *the mRS of five patients were unavailable COVID-19: coronavirus disease 2019; TIA: transient ischemic attack; MI: myocardial infarction

Variable	Values
Gender, n (%)	
Male	12 (66.7)
Female	6 (33.3)
Nationality, n (%)	
Saudi	9 (50.0)
Non-Saudi	9 (50.0)
Known comorbidities, n (%)	
Diabetes mellitus	16 (88.9)
Hypertension	13 (72.2)
Prior stroke, TIA, or MI	5 (27.8)
Kidney diseases	8 (44.4)
COVID-19 severity, n (%)	
Asymptomatic or pre-symptomatic	1 (5.6)
Mild illness	1 (5.6)
Moderate illness	3 (16.7)
Severe illness	7 (38.9)
Critical illness	6 (33.3)
Required mechanical ventilation, n (%)	13 (72.2)
Location of stroke, n (%)	
Middle cerebral artery (MCA)	9 (50.0)
Posterior cerebral artery (PCA)	3 (16.7)
Basilar artery	3 (16.7)
Deep/lacunar	1 (5.6)
MCA and PCA	1 (5.6)
MCA, PCA, and anterior cerebral artery (ACA)	1 (5.6)
Lesion side, n (%)	
Bilateral hemispheres	2 (11.1)
Left hemisphere	6 (33.3)
Right hemisphere	9 (50.0)
Midbrain and pons	1 (5.6)
Modified Rankin Score (mRS), n (%)*	
0-2	3 (16.7)
3-5	1 (5.6)
6	9 (50.0)
Outcomes, n (%)	
Alive	3 (16.7)
Dead	11 (61.1)
Referred to another center	4 (22.2)

**Figure 3:**
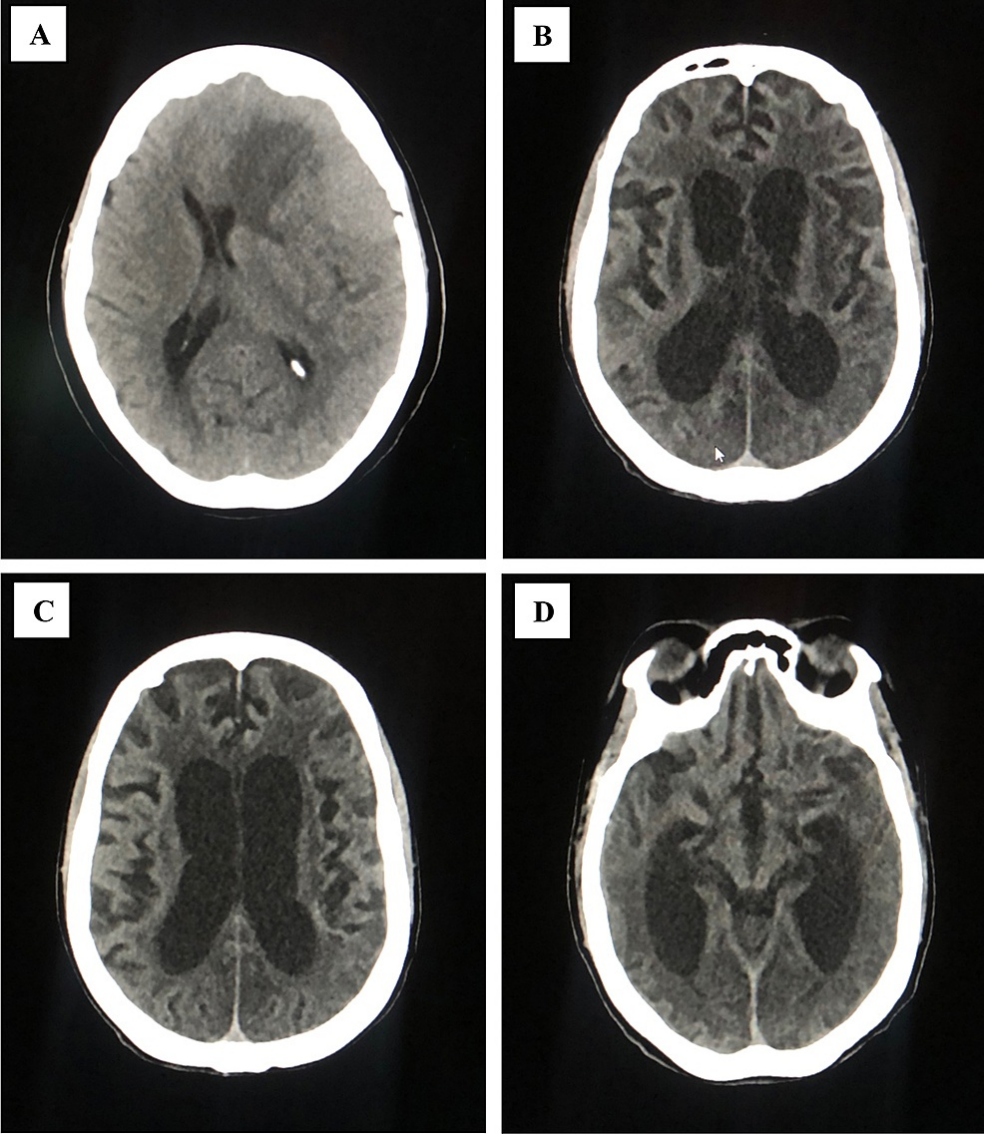
Non-contract CT scan of the brain of two patients with COVID-19 who had acute cerebrovascular complications (A) Large mass lesion. (B) Bilateral severe atrophic changes with diffuse extensive white matter hypodensities. (C) Loss of gray/white matter differentiation in the left parietal region. (D) Bilateral occipitotemporal loss of gray/white matter differentiation CT: computed tomography; COVID-19: coronavirus disease 2019

A brain CT scan of one of the included patients with ischemic stroke showed interval development of severe atrophic changes with diffuse extensive white matter hypodensities and bilateral parieto-occipital and left temporal loss of gray/white matter differentiation. Bilateral basal ganglia vanishing was also noticed. The patient’s condition could be related to diffuse hypoxic-ischemic injury. Figures 3B, 3C, 3D show the images of the non-contrast CT brain underwent by the patient.

One patient had a mass lesion that was detected on the same day of admission after the confirmation of COVID-19 infection. Figure 3A is an unenhanced CT scan of the brain that shows a large isodense mass lesion (9.1apx6.1trx4.9cc CM) in the left fronto-temporoparietal region with dense foci of calcification and a few cystic changes seen within the lesion. The mass lesion was surrounded by the adjacent parenchyma edema with a midline shift of 9.8 mm towards the right side. The patient underwent a contrast MRI for further evaluation.

Factors associated with stroke in COVID-19 patients 

Table 4 presents the model that includes all the shown risk factors, among which hospitalization period, DM, respiratory failure, ARDS, and critical illness were the significant risk factors, keeping all other factors constant. Staying at the hospital for one to two weeks was associated with 81% less likelihood for stroke compared to staying for less than one week (OR=0.19; 95% CI: 0.04-0.95). Also, staying for more than two weeks was associated with 88% less risk for stroke compared to staying for less than one week (OR=0.12; 95% CI: 0.2-0.92). Diabetic patients with COVID-19 infection had nearly four times the risk for a stroke compared to non-diabetics (OR=3.76; 95% CI: 1.1-29.9). Also, patients with respiratory failure had 21 times more likelihood to have a stroke compared to those without (OR=21.3; 95% CI: 2.2-54.6). COVID-19 patients with ARDS recorded a three-fold increased risk for developing stroke than those without (OR=2.96; 95% CI: 1.25-37.3). Also, critically ill patients had nearly double the risk for stroke than asymptomatic cases (OR=1.8; 95% CI: 1.1-6.9).

**Table 4:** Exact logistic regression model for factors associated with stroke among COVID-19 patients *Statistically significant COVID-19: coronavirus disease 2019; OR: odds ratio; CI: confidence interval; TIA: transient ischemic attack; MI: myocardial infarction; ARDS: acute respiratory distress syndrome

Factors	OR_A_	95% CI		P-value
		Lower	Upper	
Age (years)				
<60	Reference			
60-70	2.16	0.37	12.70	0.396
>70	0.68	0.06	8.09	0.756
Gender				
Female	Reference			
Male	0.73	0.13	3.94	0.711
Nationality				
Non-Saudi	Reference			
Saudi	0.50	0.08	3.13	0.458
Hospitalization period (weeks)				
<1	Reference			
1-2	0.19	0.04	0.95	0.043*
>2	0.12	0.02	0.92	0.041*
Diabetes mellitus	3.76	1.01	29.95	0.047*
Hypertension	1.20	0.07	2.11	0.267
Prior stroke, TIA, or MI	0.56	0.08	4.10	0.568
Respiratory failure	21.30	2.18	54.61	0.040*
Septic shock	1.60	0.26	9.69	0.609
ARDS	2.96	1.25	37.32	0.042*
COVID-19 severity				
Asymptomatic	Reference			
Mild to moderate	0.19	0.00	7.98	0.382
Severe	0.71	0.02	25.97	0.853
Critical illness	1.80	1.10	6.90	0.039*

## Discussion

From a total of 1,661 patients with a confirmed diagnosis of COVID-19, we included 79 patients who had a neurological manifestation during the study period. The most frequently reported neurological symptoms were altered level of consciousness, dizziness, and focal neurological deficit. Acute ischemic stroke was the most common finding on neuroimaging, and the most affected site was the right MCA territory. The most significantly associated risk factors that increased the risk of having a stroke were DM, respiratory failure, ARDS, and being critically ill. Other neurological complications observed were hemorrhagic stroke, subacute/chronic infarction, meningitis, and brain mass lesion.

Pathophysiology of the neurological features in COVID-19 infection

SARS-CoV-2 is an enveloped, non-segmented, single-stranded, positive-sense RNA virus that belongs to the beta-Coronaviridae family [18-20]. Some hypotheses suggest that the neurotropism of SARS-CoV-2 is similar to that of previous coronaviruses: SARS-CoV-1 and MERS-CoV. The route of SARS-CoV-2 invasion is through the expression of angiotensin-converting enzyme 2 (ACE2) receptor on blood-brain barrier endothelial cells, enabling viral entrance into the central nervous system. This direct route of invasion causes neurologic damage to particular receptors and retrograde travel along nerve fibers [21,22], while the indirect mechanism includes the activation of the global systemic inflammatory response syndrome (SIRS) generated by the binding of SARS-CoV-2 at the pulmonary epithelial cells, which causes cytokines storm [23]. These effects localize alveolar injury result in hypoxia, which in turn leads to cerebral edema and ischemia [2,23].

Neurological signs and symptoms associated with COVID-19 infection

As shown in Table 2, the current study found that the most frequent neurological manifestation among the 79 COVID-19 patients is altered level of consciousness (45.9%), followed by dizziness, focal neurological deficit, headache, and syncope/fall (11.5%, 10.4%, 4.2%, and 1% respectively). This finding is contrary to a previous systematic review of neurological symptoms and complications of COVID-19, which found that headache was the most reported manifestation in 51 studies [3,308/16,446 (20.1%)], ranging from 2.0% to 66.1%, while dizziness was the most reported one in 13 studies [151/2,236 (6.8%)], ranging from 2.5% to 21.4%, and the least reported manifestation was impairment of consciousness [146/2890 (5.1%)], ranging from 1.4% to 69.0% [24]. This difference in values related to frequent manifestations may suggest that these signs and symptoms may develop due to indirect mechanisms of neuropathogenicity such as hypoxia, respiratory distress, COVID-19 severity, and other comorbidities [24,25].

Neurological complications associated with COVID-19 infection

Severe neurological complications of COVID-19 such as encephalopathy and stroke have been seen not uncommonly in hospitalized COVID-19 patients. Other pathologies with direct brain invasion such as encephalitis meningitis and viral detection in the cerebrospinal fluid (CSF) have been reported less frequently [21]. Our results showed three cases with positive neuroimaging consistent with meningitis, although the CSF samples were not conclusive. Nevertheless, several reports with a positive CSF sample for SARS-CoV-2 have been published [26-29]. In cases of encephalopathy, it can be caused by both a systemic dysfunction (systemic encephalopathy) or direct neuro-infiltration (encephalitis). And the only way to establish a correct diagnosis is either by MRI, a positive CSF sample, RT-PCR, or an electroencephalogram (EEG) [30].

Regarding stroke, Iadecola et al. have described in a systematic review that the literature reported a rate reaching an upward value of 1-3% [21]; moreover, Jain et al. have reported that acute stroke is the most common finding in neuroimaging in hospitalized COVID-19 patients [8]. These reports are consistent with our finding that 1.14% (19/1,661) of all patients were diagnosed with a stroke. Multiple pathophysiological etiologies of stroke in COVID-19 have been hypothesized, such as coagulopathy, cardiac injuries, cardiac arrhythmias with subsequent cardiac embolism, secondary bacteremia, and septic emboli (28). We also reported a significant statistical association of multiple risk factors such as hospitalization period of more than one week, DM, respiratory failure, ARDS, and critical illness with stroke; surprisingly, this statistical association was not found with age. However, it could not be determined that if stroke development is due to COVID-19 as a cause or due to the presence of other comorbidities. Patients who developed stroke and had positive neuroimaging findings had a mortality rate of 55.6%, which is consistent with a radiological study done on COVID-19 patients that reported a mortality rate of 50% [8].

Limitations

This study has several limitations. Firstly, bias related to the retrospective nature of the study could be possible since our data were collected from the reports in patient files. Missing data due to lack of documentation or recall bias is an unavoidable limitation of a retrospective study of this nature. Secondly, some patients were referred to other hospitals, and information regarding their clinical outcomes was unavailable. Additionally, data were collected in a short timeframe and from a single center. However, despite these limitations, we have tried to ensure the maximum reliability of the collected data. Our inclusion criteria were based on both an acute neurological symptom and a recent brain imaging, which made the diagnosis of our cases very reliable. We believe that further multi-center, long-term prospective studies might be required to provide more insights into the long-term neurological consequences of COVID-19.

## Conclusions

It has been shown that neurological symptoms and complications are not uncommon among COVID-19 patients. COVID-19 patients might present with a wide range of neurological symptoms. Our study revealed that altered level of consciousness, dizziness, and focal neurological deficit were the most frequently reported neurological symptoms among COVID-19 patients at the Al-Noor Specialist Hospital in Makkah, Saudi Arabia. Acute ischemic stroke was the most common finding on neuroimaging, and it was significantly associated with DM, respiratory failure, ARDS, and being critically ill. Further studies are required to provide more data and knowledge about this issue, including the possible risk factors and the long-term neurological consequences of COVID-19.
